# A Comparison of Prices Paid by London Hospitals for Their Food Supplies.—I

**Published:** 1912-04-06

**Authors:** 


### INSTITUTIONAL HOUSEKEEPING.

**(Workers' Questions and Criticism of every kind will be welcomed.)**

### A Comparison of Prices Paid by London Hospitals for their Food Supplies.—I.

Considerable amazement, not to say amusement, was caused by the publication in the statistical report of the King's Fund, of a "table showing the highest and the lowest of the prices returned as the lowest which were being paid on December 31, 1910, for forty-six different articles in use by 105 of the hospitals dealt with in the Report."

We have submitted the table to an expert buyer who has had long experience of the markets, and we think that his report will be read with much interest. It must be borne in mind that the prices quoted are in all cases, whether high or low, "the lowest which were being paid" in some institutions. The lamentable ignorance of buying which characterises many of those responsible for victualling great establishments supported by voluntary subscriptions is forcibly illustrated by these figures.

At the same time we would call attention to defects in presentation, which spoil the value of the figures. The double meaning attaching to the gallon measure of milk should have been guarded against. The word "sugar" is far too vague, and "bacon" may mean any part of the pig.

*Topsides of Beef.*—Highest, 10d.; lowest, 3d. per lb. The highest price paid, 10d., should command quite a first-class article, joints cut from English or Scotch quarters; but some error would appear to have been made in quoting the minimum price, 3d. A very advantageous contract made in 1910 may possibly have secured the lowest class of frozen beef at an all-round price of 3d. per lb., taking fore and hind quarters in equal proportion, but topside being one of the prime parts of the bullock, a fair estimate of its cost, taking into consideration the much smaller value of many of the parts, would not even in that case be less than 5d. At the present time it would be difficult to make a contract for frozen topside at less than 5¾d.

*Legs of Mutton.*—Highest price, 9½d.; lowest price, 4d. per lb. Here again there are wide differences in quality, but 9½d. is a fair price for good quality English or Scotch meat, whilst the minimum, 4d., is not too much to pay for frozen legs of fair quality, the latter being probably Australian or River Plate.

*Fish.*—Highest price, 6d.; lowest price, 1½d. per lb. Apparently the varieties used in hospitals are not those which come under the category of "prime." Neither soles, turbot, brill, halibut, salmon, nor any fish of similar kind could be purchased at the maximum price of 6d. per lb., whilst the minimum price, 1½d., could only represent the very commonest kinds of coarse fish. In the absence of information as to the actual varieties received for the prices it is impossible to say whether too much has been paid. Plaice, cod, haddocks, whiting, etc., should, however be obtainable for 6d. per lb. or less, except during very stormy weather or at other times of exceptional shortage.

*Fowls.*—Highest price, 4s.; lowest price, 1s. 9d. each. There is a wide difference between the maximum and the minimum prices, but neither is extraordinary. Russian chickens are obtainable at most seasons of the year at 1s. 9d., whilst large-sized Surrey fowls are always worth 4s. each and even more at certain seasons; and at intermediate prices supplies can be obtained all the year round, varying in size and quality according to the cost. For a year's contract good-sized Lincolnshire fowls should be obtainable at from 2s. 6d. to 2s. 9d. each.

*Bacon.*—Highest price, 1s.; lowest price, 5½d. per lb. The average price for sides of good Irish or Danish bacon is 8d. per lb. Therefore, presuming 1s. to be the price paid for the primest parts, for instance small quantities of back or gammon rashers, 1s. per lb. is not dear; but this figure is far too high for a contract for bacon in any quantity, whilst a contract could only be made for the coarsest parts, such as hocks and flanks, at the minimum price of 5½d.; it would be impossible in any circumstances to obtain prime parts for this figure.

*Butter.*—Highest price, 1s. 2d.; lowest price, 10½d. per lb. The maximum price, 1s. 2d., is a fair average one for butter of good class, such as Danish, Colonial, or Normandy, whilst at 10½d. only the poorest of Siberian could be bought at any time. There is apparently no extravagance in this department.

*Eggs.*—Per 120: New laid—Highest price, £1; lowest price, 8s. 6d. Cooking—Highest price, 14s. 6d.; lowest price, 7s. 6d. 8s. 6d. per 120 is a ludicrous price for new laid eggs to include the winter season, and even 20s. could not at that time of the year command English eggs in such quantities as would be required by a large hospital. Cooking eggs at 7s. 6d. per 120 would also at that season probably be of the electioneering variety. In mid-winter good-class cooking eggs cannot be got at less than 11s. 6d. per 120, and 14s. 6d. is not too much for large sized eggs of good quality.

*Milk.*—Highest price, 1s. 10d.; lowest price, 7¾d. per gallon. Before a comparison of prices can be made it is desirable to ascertain whether the quotations are in each instance for the same measure. The ordinary gallon contains eight pints, but in the wholesale milk trade "barn measure" is employed, in which case the gallon contains sixteen imperial pints. Again the quotation is simply for "milk," and may therefore apply to either new or skimmed. At the present moment the wholesale price of new milk is from 2s. 4d. to 2s. 6d. per barn gallon, but perhaps in 1910 it was just possible to make a contract at 1s. 10d. If, however, 1s. 10d. represents the price for an imperial gallon a large margin of profit must have accrued to the vendor. The minimum of 7¾d. was no doubt the price paid for skimmed milk, this being about the value of that article in the winter months.

